# Development of ELISA-based methods to measure the anti-malarial drug chloroquine in plasma and in pharmaceutical formulations

**DOI:** 10.1186/1475-2875-10-249

**Published:** 2011-08-24

**Authors:** Insaf F Khalil, Michael Alifrangis, Camilla Recke, Lotte C Hoegberg, Anita Ronn, Ib C Bygbjerg, Claus Koch

**Affiliations:** 1 Centre for Medical Parasitology at Department of International Health, Immunology and Microbiology, University of Copenhagen and Department of Infectious Diseases, Copenhagen University Hospital, CSS Oster Farimagsgade 5, 1014 Copenhagen K, Denmark; 2 Bio Port A/S/. Grusbakken 8, DK-2820 Gentofte, Denmark; 3 Department of Toxicology, Bispebjerg Hospital, Bispebjerg Bakke 23 2400 Copenhagen NV, Denmark; 4 Institute of molecular medicine, Department of Cancer and Inflammation, University of Southern Denmark, DK-5000 Odense

## Abstract

**Background:**

In Central and South America and Eastern and Southern Africa, *Plasmodium vivax *infections accounts for 71-81% and 5% of malaria cases, respectively. In these areas, chloroquine (CQ) remains the treatment of choice for *P. vivax *malaria. In addition, CQ has recently proven to be an effective HIV-1 therapeutic agent. There is a dire need to continue monitoring quality of CQ as there is a major influx of substandard and fake formulations into malaria-endemic countries. The use of fake/substandard drugs will result in sub-therapeutic levels endangering the patient and possibly select for parasite resistance. The aim of this study was to develop an inexpensive, simple antibody-based ELISA to measure CQ concentrations in tablets and in plasma.

**Methods:**

A monoclonal antibody (MAb) that reacts with the N-side chain of the CQ molecule was prepared by use of a CQ analogue. A specific and reliable ELISA for detection of CQ was developed. The developed assay was validated by measuring CQ in tablets sold in Denmark, India and Sudan. Furthermore, kinetics of CQ concentrations in plasma of four volunteers, who ingested two tablets of Malarex^® ^containing, 250 mg CQ base, were measured before drug intake, three hours later and thereafter at days 1, 3, 7, 14, 21 and 28. The same plasma samples were simultaneously measured by high performance liquid chromatography (HPLC).

**Results:**

The ELISA proved an easy-to-handle and very sensitive tool for the detection of CQ with a lower limit of detection at 3.9 ng/ml. ELISA levels of CQ in plasma showed high agreement with the levels obtained by HPLC (r = 0.98). The specificity in the negative control group was 100%.

**Conclusion:**

The developed ELISA can be used for quality screening of CQ in pharmaceutical formulations and for drug monitoring in malaria and in other infectious diseases, such as HIV, where CQ proved to be an effective therapeutic agent. The methodology has been exploited to develop monoclonal antibodies for the drugs used in artemisinin-based combination therapy (ACT).

## Background

Malaria-associated morbidity and mortality both in children and adults is reported in many tropical countries. *Plasmodium vivax *accounts for over half of all malaria transmitted outside Africa. The development and spread of drug resistance in malaria parasites has spurred on a global change in policy from the use of the former first-line anti-malarials chloroquine (CQ) and sulphadoxine/pyrimethamine (SP) to the use of artemisinin-based combination therapy (ACT) for uncomplicated malaria. However, CQ is still the first-line treatment for *P. vivax *malaria in most parts of the world [1-3].

Ensuring that the ACT reaches the majority of children and vulnerable adults in need has proved challenging and the reality on the ground is a continued use of CQ and SP in private and public facilities [4]. CQ continues to be widely used in Madagascar, despite having been officially replaced by ACT for treatment of uncomplicated falciparum malaria in 2005 [5]. Prepackaged CQ is still recommended by the Ministry of Health and Family Planning for the home management of presumed malaria in children under the age of five years [5].

Marketing of fake/substandard CQ has been widely reported in malaria endemic areas with a weak drug regulatory system [6-10]. The intake of fake medicines can have life-threatening consequences, such as patients suffering or death and numerous adverse effects due to ineffectiveness, under-dosing, over-dosing, unexpected or toxic substances [6,8]. Moreover, it will influence the economic welfare of patients as treatment has to be repeated several times and consequently patients will lose trust in health systems [6]. Finally, use of substandard CQ will most

likely increase risk of selection and spreading of drug resistance to the structurally and/or functionally related ACT partners currently in use, including amodiaquine [8,9].

Thus, there is an urgent need to invest in developing simple, quick, inexpensive and sensitive methods to measure levels of active drugs in pharmaceutical formulations at point-of-purchase and in patient's plasma in clinical trials that monitor susceptibility of *P. vivax *to CQ. Current methods for quantification of CQ in plasma and in pharmaceutical formulations include fluorimetry, gas liquid chromatography, high performance liquid chromatography (HPLC) and radio-immunoassays [11-14]. Even though the chromatographic methods offer the required sensitivity, reliability and specificity, they require expensive and complex equipment as well as highly trained staff. Their use is thus restricted to well-equipped laboratories, which may often not be accessible in malaria-endemic areas.

ELISA assays using poly or monoclonal antibodies (MAb) against CQ have been developed, but with low specificity for CQ as they recognize all compounds bearing the 4-aminoquinoline moiety, in particular CQ's main metabolite, desethyl CQ, amodiaquine and its metabolite desethyl amodiaquine because the antibodies have been raised against the quinoline group [15-18]. A specific ELISA has been developed for detection of CQ in urine and blood spotted on filter paper by raising polyclonal antibodies in sheep against CQ conjugated to keyhole limpet haemocyanin immunogen [19].

The aim of this study was to develop field applicable ELISA methods for estimating CQ levels in clinical specimens and in tablets through the production of high-affinity monoclonal antibody against the N-side chain of CQ. The monoclonal antibody was developed in collaboration with University of Southern Denmark and the private company Bio Port A/S, Denmark. The procedure resulted in a monoclonal antibody designated HYB 317-01. The antibody is commercially available.

## Methods

CQ (purity 98%), 2-amino-5-diethylaminopentane (ADP) and bovine serum albumin (BSA) were provided by Sigma-Aldrich (Denmark). The carrier protein, S3 (secreted proteins from cultures of Bacillus-Calmette-Guerin) and Bacillus-Calmette-Guerin (BCG) vaccines were kindly provided by The State Serum Institute (Denmark). Polyclonal anti-mouse immunoglobins/HRP P0260 antibody and OPD were purchased from Dako (Denmark). All other chemicals were standard commercial products of analytical grade.

### Development of the monoclonal antibody

#### Preparation of the conjugate

**Figure 1:**
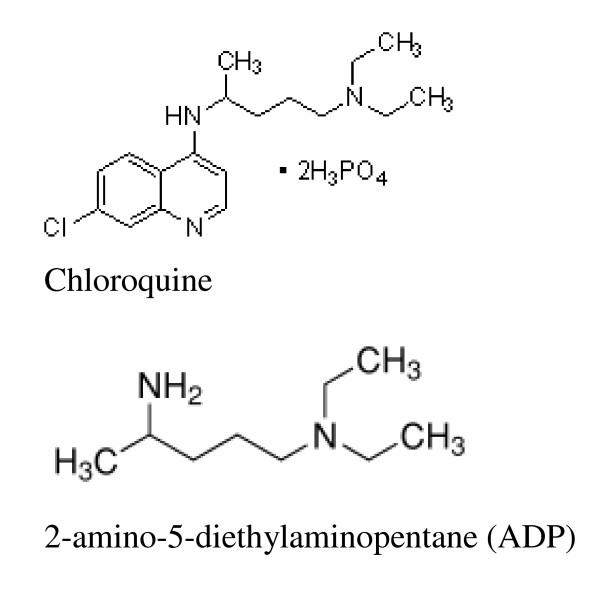
**The chemical structure of chloroquine and the 2-amino-5-diethylaminopentane (ADP)**.

The hapten 2-amino-5-diethylaminopentane (ADP) which corresponds to the N-side-chain of the 4-amino-7-chloro-quinoline moiety of the CQ molecule (Figure 1) was used for coupling to the immunogenic carrier protein S3. A five-fold molar excess of ADP was reacted with the protein using a standard glutaraldehyde coupling procedure [20].

#### Immunization and hybridization

Mouse experiments have been performed according to permission 561-1758 from the Danish Animal Experiments Inspectorate. Three months old female CF1xBALB/c mice were pre-immunized with 0.2 ml BCG vaccine 4 weeks prior to administration of the ADP-S3. ADP-S3 was adsorbed onto Alhydrogel (Brenntag Bio-sector, DK). Immunization doses of 25 μg ADP-S3 containing 1 mg Al(OH)_3 _were given i.p. four times with two week intervals (Claus Koch, personal communication). Boost vaccination before fusion was done i.v with 25 μg ADP-S3.

Three days after the i.v boosting, spleens were removed from two mice with high titres of antibodies to CQ, and the spleen cells were pooled and mixed with myeloma cell line X63Ag8.653. Hybridoma cell lines were prepared by fusing the cells using PEG [20]. The fused cells were cultured in enriched RPMI 1640-Dulbecco's-Ham F12 medium containing an aminopterin to selectively obtain hybridomas. The hybridoma culture supernatants were screened by an ELISA in which ADP-ovalbumin was used as solid phase antigen after cell fusion. Furthermore, a competitive ELISA in which CQ (50 μg/ml) was used as free antigen was also performed to select hybridomas producing a monoclonal antibody with a specific reactivity to CQ. The selected hybridomas were cloned twice with the limited dilution method and cultured on a large scale.

#### Purification of the monoclonal antibody

The produced monoclonal antibody was purified using a Protein GFF column (0.46 × 11 cm; Pharmacia Biotech; Uppsala, Sweden). The culture medium (700 ml) containing the IgG was adjusted to pH 7 with 1 M Tris solution and subjected to the column. The column was washed with 10 mM phosphate buffer (pH 7). Adsorbed IgG was eluted with 100 mM citrate buffer (pH 3). The eluted IgG was neutralized with 1 M Tris solution and then dialyzed against water five times. The isotype of anti-CQ monoclonal antibody was classified by using a mouse ID Kit (Cat no. 90-6550) from Zymed.

### Preparation of standard reference solution and samples used in the antibody-based competitive ELISA

#### Stock solutions and calibration standard

Stock solution of CQ was prepared in deionised water at a concentration of 1 mg/ml. Working solutions were prepared by appropriate dilution of the stock solution in the ELISA dilution buffer (PBS + 0.37 M NaCl + 1% BSA + 0.05% Tween 20, pH 7.2). These working solutions were used to prepare seven calibration standards in the range 0.1-2000 ng/ml. The standards were either prepared in the dilution buffer for analysis of CQ tablets or spiked in plasma for detecting CQ in plasma samples.

#### Competitive ELISA

For highly reliable results, checker board ELISA using varying dilutions of the coating antigen (ADP-OA) and anti-CQ HYB 317-02 was performed to determine the optimal concentrations for the inhibition assay, at which colour intensity was around 2.0. Afterwards competitive inhibition assay was developed and validated. ADP: OA (0.1 μg/ml) was used as coating antigen. One hundred μl of the diluted antigen (1:400 in 0.05 M carbonate buffer at pH 9.6) were added to each well and incubated overnight at 4°C in moist boxes. After incubation the plates were washed four times in washing buffer (PBS with 0.5 M NaCl + 0.1% Triton-X100, pH 7.2). 100 μl of each sample and CQ monoclonal antibodies (1:2500 in the dilution buffer) were incubated for an hour at room temperature and afterwards 100 μl were added to the microtitre plates, final concentration of monoclonal antibody in the samples was 1:5,000. Test plates were incubated for 1 hour on a shaker at RT, and then washed 4 times as before. 100 μl of HRP P0260 (1:1,000) were added to each well and the plates were incubated for 1 hour at RT. Finally, the plates were washed 4 times and 100 μl of the substrate-solution (OPD 4 mg/ml in water and 0.4% hydrogen peroxide), were added and the plates were incubated for 12 minutes and the reaction was stopped using 1.0 N H_2_SO_4_. The colour intensity of the solution was read in a microtitre reader at 490 nm wavelength.

### Validation of the ELISA assay

#### Specificity of the developed monoclonal antibodies

Specificity and cross-reactivity was defined by the competitive affinity of the monoclonal antibody for the related 4-aminoquinolines and their metabolites; DCQ, AQ and DAQ, hydroxyl-chloroquine, mefloquine, quinine, pyrimethamine, trimethoprim, sulphadoxine and sulphamethoxazole.

#### Linearity

Linearity of the ELISA method was tested over the range 0.1 - 500 ng/ml.

#### Precision, accuracy and analytical recovery

The intra-run and inter-run variation from plate to plate were tested using a high (300 ng/ml), a middle (150 ng/ml) and a low level of CQ (75 ng/ml) from the calibration standards and run in triplicate in three plates under the same conditions in the same day and over six days. The coefficient of variation (%CV), accuracy and analytical recovery were then determined at each level.

#### Lower limit of detection (LLD) and limit of quantification (LLQ)

LLD was defined as negative control ± 3 SD above the mean OD for the 1:2 dilutions of plasma samples collected from healthy Danish volunteers with no history of CQ intake. The LLQ was determined by testing replicates of low concentrations (25-0.1 ng/ml) of CQ spiked in the dilution buffer or in plasma.

### Applicability of the inhibition ELISA

#### Test of CQ concentration in tablets

The feasibility of the method was demonstrated by measuring of CQ concentrations in pharmaceutical formulations manufactured in Denmark (Malarex^® ^and Ercoquin^®^), Sudan (Eliquine^®^) and India (Chloroquine from Vikram Laboratories). CQ content per each tested tablet was 250 mg. The tested drugs were purchased from pharmacies in quantities that contain 10 tablets. Efforts were made to ensure that the drugs were not degraded by inadequate storage conditions, which would have confounded the findings. The drugs were stored for 4.5 months before the analysis at room temperature and protected from sunlight and humidity. None of the analysed drugs had expired upon analysis.

The amount of the active ingredient as declared by the manufacturer was noted. Three samples were separately analysed. In each run, three tablets of CQ were weighed and finely pulverized. Then a proportion of this powder corresponding to the average weight of a tablet were accurately weighed and transferred to 100 ml volumetric-flask containing methanol: glacial acetic acid (100:2%, respectively) for complete dissolution to achieve a concentration of 2.5 mg/ml. This solution was shaken vigorously and filtered. Three different concentrations (50, 100 and 200 ng/ml CQ) were made in the ELISA dilution buffer and they were ran a long in inhibition ELISA. Drug quality was assessed by comparing the amount of active ingredient (CQ) in the eluents of each dissolution sample against a known concentration of standard CQ in the rage 0.1 - 500 ng/ml.

#### Test of CQ concentration in plasma samples

Plasma samples were donated by four volunteers, who ingested a single dose of two tablets of Malarex ((Dumex Ltd, Copenhagen, Denmark) that contains 250 mg CQ base/tablet. The total amount of CQ given to the volunteers was equivalent to 500 mg. Blood samples were taken before drug intake, three hours thereafter and at days 3, 7, 14, 21, and 28 post-treatment. The samples were stored in dark at -20°C. Fifty μl of each sample were tested by both high performance liquid chromatography (HPLC) and the competitive ELISA to validate the developed method. The values of CQ concentrations obtained by both methods were compared.

#### HPLC

A reverse phase HPLC with UV was used as the gold standard for the developed ELISA. In the assay 50 μl plasma samples diluted with 90 μl of water and alkalinised with 200 μl carbonate buffer (0.1 M, pH 9.5), were extracted into 2 ml *tert*-butylmethylether. The drug was thereafter re-extracted into an acidic aqueous phase with 150 μl phosphate buffer (0.1 M, pH 4.0). The HPLC column was a Zorbax SB-CN (5 mm, 4.6 ID × 250 mm) protected by a LiChroCART^® ^4-4 LiChrospher^® ^100 CN (5 mm) guard column, and used with a mobile phase consisted of acetonitril: phosphate buffer (0.1 M, pH 2.3):sodium perchlorate (1.0 M); (13:86:1, v/v). The flow rate was 1.2 ml/min. and the absorbance of the drugs was measured at 237 nm.

## Results

Hyper-immunization of BALB/c mice with ADP-OA-S3 yielded the monoclonal antibody HYB 317-02. The antibody was purified by G affinity chromatography in order to develop a reliable ELISA to measure CQ in tablets and in biological fluids. The isotype of anti-CQ HYB 317-02 was classified as having κ light chain of the IgG2b subclass antibody group. The optimal concentrations of the antigen and anti CQ monoclonal antibody concentrations to be used for the inhibition assay were found to be 1:400 and 1:2500, respectively.

### The standard curve

**Figure 2:**
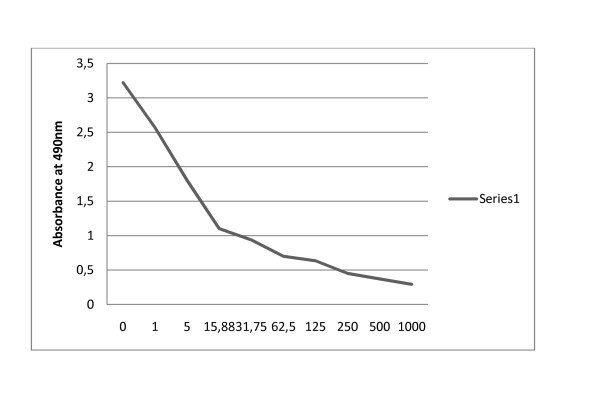
**Calibration curve for determination of chloroquine concentration under the conditions used for estimation of precision, recovery and accuracy**.

The full measuring range of the developed assay for CQ extends from 0.1 to 500 ng/ml. Figure 2 shows a graph that represents a typical calibration curve under the optimal conditions used for the determination of specificity, precision, accuracy and recovery.

### Specificity, sensitivity and resolution

**Figure 3:**
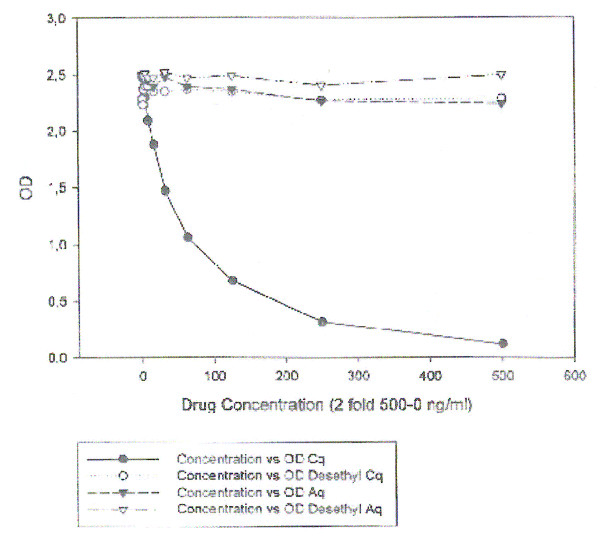
**Cross-reactivity between chloroquine, desethyl chloroquine, amodiaquine and desethyl amodiaquine in plasma samples**.

The monoclonal antibody was specific for CQ with no cross-reactivity to the related aminoquinolines over a concentration range of 10 - 500 ng/ml (Figure 3). The monoclonal antibody does not cross-react with mefloquine, quinine, sulphadoxine, sulphamethoxazole, trimethoprim or pyrimethamine in the above mentioned concentration range. The ELISA system was highly sensitive with a LLD and LLQ of 0.1 and 3.9 ng/ml, respectively. Endogenous materials in plasma or in CQ tablets did not interfere. Within, between assay coefficients of variation (CV) and analytical recovery are shown in Table 1. To minimize variations due to evaporation of the reagent, and uneven temperature during incubation from day to day, a fresh standard curve was used with each run. Both intra and inter-assay CV were < 10% at all tested levels (Table 1).

**Table 1:** Precision and analytical recovery of a high, medium and low concentrations of CQ in plasma

Parameter	Low concentration 75 ng/ml	Medium concentration 150 ng/ml	High concentration 300 ng/ml
Within assay CV%	6.5	6.9	5.7
Between assay CV%	6.8	6.8	6.2
Analytical recovery	92.2%	93.8%	93.4%

CV: Coefficient of variation.

### Determination of CQ in tablets and in plasma

**Figure 4:**
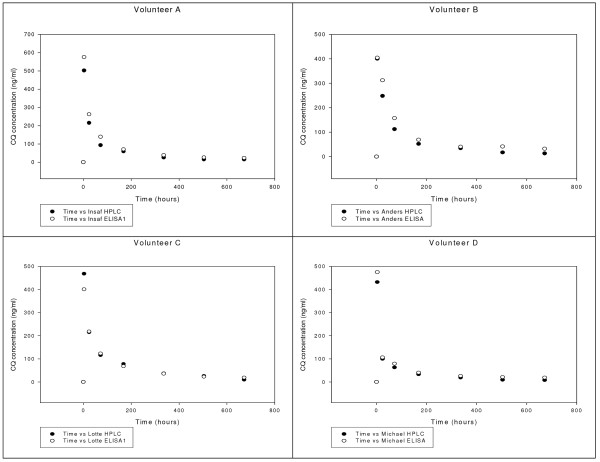
**Chloroquine concentration measured by HPLC and the inhibition ELSA using CQ MAb HYB 317-02 in 4 volunteers after an ingestion of a single dose of 500 mg CQ base**.

**Figure 5:**
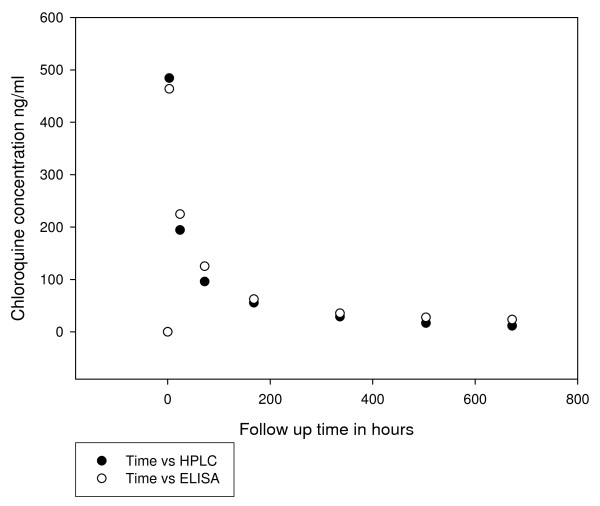
**Chloroquine concentrations measured by HPLC and the inhibition ELISA**.

HPLC was used as a reference method in assessing the ELISA technique. The accuracy of the method ranged from 92 to 98 at all tested concentrations (10 - 2000 ng/ml) and the recovery of CQ in the therapeutical formulations was found to range from 95-96% (Table 2). The percentage of the active ingredient (CQ) in the four formulations tested in this study was found to be between 98-104%. Plasma concentrations of CQ determined by both ELISA and HPLC for the four volunteers are shown in Figure 4. There was a good correlation (r ranged from 0.96-0.99) and essentially identical results were obtained by both analytical methods (Figure 5).

**Table 2:** Precision and analytical recovery of CQ in pharmaceutical formulations

Nominal concentration (ng/ml)	Recovery (%)	Precision (%RSD)
200	97,2	3.1
100	95.0	5
50	95.0	5.4

## Discussion

CQ (Figure 1) is poorly immunogenic because of its low molecular weight. For the production of a sufficient amount of antibody against CQ, the hapten ADP (Figure 1) was conjugated to ovalbumin (OA) and the carrier protein S3. The hapten serves as an epitope for binding to the antibodies on the B-cell surface and the carrier protein provides class II T-cell receptor binding sites necessary for T helper cell activation and subsequently for antibody production. The hapten which is an analogue to the N-side chain of CQ was chosen for the production of a monoclonal that could not cross-react with the other aminoquinoline drugs, as illustrated in Figure 2. The hapten was easily linked to the bi-functional linker glutaraldehyde that provides covalent bonds to the amino groups in the hapten and in the carrier protein. The bridge formed by glutaraldehyde could be recognized as an epitope by subsequent immunization.

Validation of the ELISA shows that the developed technique could discriminate CQ from other anti-malarial drugs, was easy-to-handle and a very sensitive tool for the detection of CQ with a lower limit of detection at 0.1 ng/ml. ELISA levels of CQ in tablets and in plasma showed high agreement with the levels obtained by HPLC (r = 0.98) and the specificity in the negative control group was 100%. However, high plasma CQ concentrations obtained in this study might be due to the facts that Blood samples were separated immediately after sample collection, plasma were kept at - 20°C in tubes treated with chlorodimethylsilane in toluene (50/o, v/v) in order to minimize drug adsorption and plasma samples were analysed two days after the collection of day 28 samples. Although the developed ELISA is as sensitive as gold standard, the HPLC, further validation in large field trial is needed.

The ELISA method has also the advantage of convenience and affordability as well as the ability to perform analysis on large number of samples in remote environments, where conditions such as temperature, humidity and reagent quality could be quite variable. It is important to include reference positive standards and negative control in each run to confirm the integrity of the assay. The technique is field-friendly and only requires an ELISA reader for quantitative measurements.

Other achievements with the present assay also include commercially available monoclonal antibody, simple extraction procedure of CQ from pharmaceutical formulations using standard organic liquids and ELISA buffers. With the developed ELISA, it took about 4 h to run a maximum of 39 patients/tablets, including nine standards and the internal controls. Only 50 μl plasma test matrix is required for the analysis. Considering only reagents, the cost of the ELISA system is roughly less than US $2 per sample.

Having these credits, the developed ELISA could be used for selection of subjects for *in vitro *sensitivity tests, where previous drug intake or/& drug pressure has to be tested and for quality screening of pharmaceutical formulations. Quality testing of CQ is done by sophisticated and expensive analytical methods that require expert personnel for both operation and maintenance. These resources are, unfortunately, not always available in the developing world, resulting in inadequate oversight of drug quality. As a consequence counterfeit and poor quality drugs which is already a huge problem, will continue to proliferate. Studies from Tanzania and Cameroon have shown that more than 38% of the tested CQ tablets had either no active ingredient, an insufficient active ingredient, the wrong ingredient, or unknown ingredient(s) [6,21]. However, in the four CQ brands, which were tested in this study the percentage of the active ingredient (98 and 104%) met the European Pharmacopoeia Content requirements of 95 - 105%.

Studies from Malawi and other countries have shown the return of CQ susceptible malaria after the drug use was abandoned [22]. This gives the possibility of using CQ for malaria prevention in the future as CQ is safe, well tolerated and has long elimination half-life. CQ could also be used in combination with other drugs with different pharmacokinetic (PK) and pharmacodynamic (PD) profiles for treatment of malaria. The developed ELISA could be used to monitor CQ quality in tablets and to identify the PK-PD profiles of CQ in trails that will identify combinations that could deter the reemergence of resistance [22].

## Conclusion

An antibody-based ELISA has been developed for determination of CQ in anti-malarial tablets and in plasma. The method is simple, fast, sensitive and accurate. The results from validation indicate that the proposed method can be applied for routine determination of CQ in plasma samples and in pharmaceutical formulations. Determination of CQ concentrations in tablets will hopefully improve quality assurance of drugs, quality of treatment and will minimize cross resistance between CQ and the ACT partners currently in use. The methodology has been exploited to develop monoclonal antibodies for ACT drugs.

## Competing interests

The authors declare that they have no competing interests.

## Authors' contributions

I K, MA, AR & IB design the study, CK supervised monoclonal antibody development, CR & I K developed the antibody, IK & MA developed and validated the CQ ELISA assay, LH did the HPLC work. IK wrote the manuscript and all authors read and approved the manuscript.
